# Supplementary material for: The interactome of intact mitochondria by cross-linking mass spectrometry provides evidence for coexisting respiratory supercomplexes
Source: Mol Cell Proteomics. 2017 Dec 8;17(2):216–32. doi: 10.1074/mcp.RA117.000470 (PMC5795388; doi:10.1074/mcp.RA117.000470)
Supplement: Supplemental Data [file supp_RA117.000470_133922_0_supp_23978_fzffwf.zip › spectra_annotation/mito_DR_spectra_annotation/201-1-1-1-21-1.pdf]

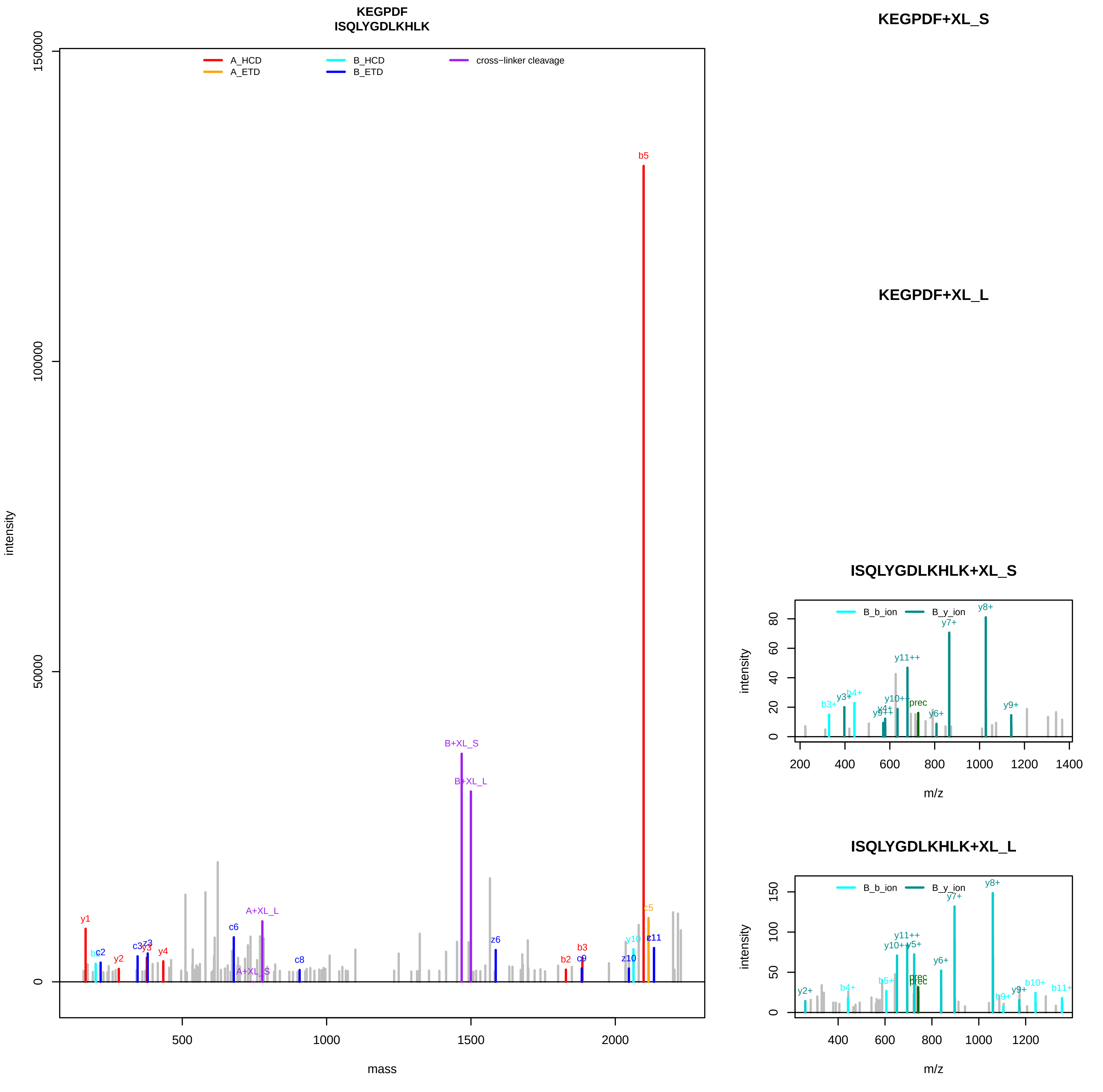

KEGPDF
ISQLYGDLKHLK
A_HCD
A_ETD
B_HCD
B_ETD
cross−linker cleavage
intensity
mass
0
50000
100000
150000
500
1000
1500
2000
b5
B+XL_S
B+XL_L
A+XL_L
A+XL_S
y1
y2
y3
y4
c2
c3
z3
c6
c8
z6
b2
b3
c9
z10
y10
c5
z11
KEGPDF+XL_S
KEGPDF+XL_L
ISQLYGDLKHLK+XL_S
B_b_ion
B_y_ion
intensity
m/z
0
20
40
60
80
200
400
600
800
1000
1200
1400
y8+
y7+
y11++
b3+
y3+
b4+
y4+
y10++
prec
y6+
y9+
ISQLYGDLKHLK+XL_L
B_b_ion
B_y_ion
intensity
m/z
0
50
100
150
400
600
800
1000
1200
y8+
y7+
y11++
y10++
y5+
y6+
b5+
prec
y2+
b4+
b9+
y9+
b10+
b11+
